# Nudging sugar portions: a real-world experiment

**DOI:** 10.1186/s40795-021-00473-9

**Published:** 2021-11-12

**Authors:** Karoline Villinger, Deborah R. Wahl, Kai Engel, Britta Renner

**Affiliations:** Department of Psychology, Psychological Assessment and Health Psychology, University of Konstanz, D-78457, P.O. Box 47 Konstanz, Germany

**Keywords:** nudging, environmental intervention, sugar overconsumption, real-world experiment

## Abstract

**Background:**

Sugar overconsumption is a major contributor to overweight and obesity, with daily consumption greatly exceeding the WHO’s recommendations. The aim of the present study was to determine whether using a functionally modified sugar shaker as a food environment nudge could be an effective means to reduce the sugar used in hot beverages.

**Methods:**

Sugar shakers were functionally modified to reduce the amount of sugar in each pour by 47%. A real-world experiment was conducted to compare the amount of added sugar per hot beverage during default and nudge conditions over the course of four weeks (17,233 hot beverages sold) in a university take-away café. In addition, 59 customers were surveyed to evaluate the acceptance of the intervention.

**Results:**

Modifying the functional design of sugar shakers resulted in a reduction of added sugar by 20% (*d* = 1.35) compared to the default condition. In the survey, most participants evaluated the intervention strategy positively.

**Conclusion:**

The present real-world experiment demonstrates that a simple environmental intervention can significantly reduce sugar consumption in public places while meeting with consumer approval, making it a promising means of reducing sugar overconsumption.

## Introduction

Sugar overconsumption poses a major health threat. Alarmingly, actual consumption of sugar greatly exceeds the WHO’s recommendation of 5 to 10% of the total energy intake [1, 2]. In this context, the way in which people sweeten hot beverages such as coffee, which is one of the most heavily consumed daily drinks in the Western world, constitutes an increasing problem [2, 3].

However, changing common daily behaviors such as sweetening coffee becomes a challenge once they are established as habits, i.e., behaviors which happen often and regularly, sometimes even without conscious awareness [4]. Nudging interventions are a promising strategy for changing habitual consumption behaviors [5] as making the desired choice the easy or default option can evoke healthier food choices (for a review, see Bucher et al. [6]).

For instance, a few observational and experimental studies indicate that a reduction of table salt use may be achieved by reducing the size or number of holes in salt shakers. In two first studies, Greenfield et al. [7, 8] reported that the amount of salt added at the table by Australian consumers in varying settings such as cafeterias or restaurants increased with the size or number of the salt shaker holes. This positive relationship between salt added at the table and area of the salt shaker hole was replicated in a canteen of a local company in the United Kingdom [9]. These finding were extended recently by Goffe and colleagues [10] who used incognito researchers for observing offered and added salt by the server in fish & chip shops in Northern England. They found that using reduced-holed salt shakers were associated with lower relative sodium content of fish and chip meals.

These results give rise to the assumption that functional modifications, i.e., modification of the ‘functionality’ (for a classification, see Hollands et al. [11]) of everyday objects might facilitate changes in habitual consumption behavior in daily life. Although the effects observed in the area of re-salting are promising, modifying the functionality of condiment containers has not yet been extensively studied. So far, there are no studies that have investigated the modification of sugar shakers and the impact on the amount of sugar added by consumers in a take-away setting.

### The present study

The present study employed a real-world experiment to investigate how effective a functional modification of sugar shakers is in reducing the amount of added sugar triggered by habitual daily behaviors such as sweetening coffee.

## Methods

The present study was approved by the University of Konstanz’s Institutional Review Board and adhered to the guidelines of the German Psychological Society and the Declaration of Helsinki.

### Design and procedures

The study was conducted over the course of four weeks. ‘Regular’ sugar shakers (default) were used in the first and third week, while in the second and fourth week they were functionally modified to release a smaller amount of sugar in each pour (nudge). ‘Regular’ sugar shakers were introduced as part of the study to allow an inconspicuous modification of the sugar shakers between study weeks. In addition, at the end of the study a subset of the customers who had added sugar to their drinks was surveyed to evaluate the study (questionnaire implemented via Qualtrics (version 1.6.4.)). All participants gave written informed consent and received a coffee voucher for participating.

### Materials and measures

#### Setting

The study was conducted at the take-away café of the University of Konstanz (Germany) which is centrally located in the main foyer of the university close to the main entrance. The take-away café is the main café of the university and represents a central meeting point for the about 10,000 students and 2300 staff of the university. Besides hot beverages, the café offers a small selection of packaged chilled drinks and small snacks (e.g. pretzel, muffin, croissant, apple, candy bar) for take-away. The café consists of a cashier’s counter for ordering and collecting drinks without any seating available. Sugar shakers are placed on a table opposite the bar together with additional milk, spoons and napkins, which customers are free to use.

#### Intervention material

**Fig. 1:**
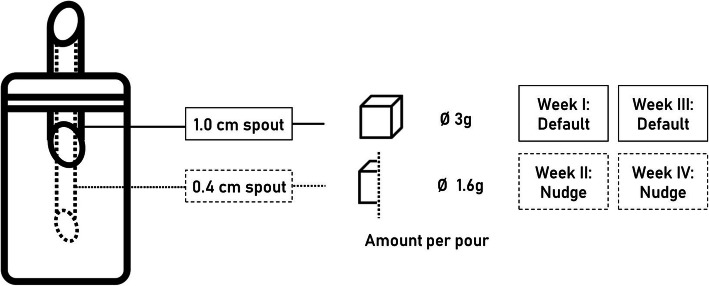
Functional modification of the sugar shakers

A common sugar shaker (Fackelmann, capacity 280 ml) was used as the default option. For the nudging condition, the diagonal of the hole was reduced from 1.0 cm to 0.4 cm by inserting a small metal pipe in the spout (see Fig. 1). To determine the amount of sugar dispensed per pour, a sugar shaker was filled with 200 g of regular granulated white sugar for each of the two conditions (default/nudge) and the average amount dispensed was measured over 25 pours. The procedure was repeated four times for each condition. The modification reduced the average amount of sugar in each pour by 47% from 2.96 g (*SD* = 0.08; default condition) to 1.58 g (*SD* = 0.07; nudge condition), which reduced the average calories per pour from 11.5 kcal to 6.1 kcal.

#### Sugar use per hot beverage

As a close proxy for sugar intake, the amount of used sugar was assessed. Each day, before the café opened, the shakers were filled with 200 g of standard granulated white sugar and in the evening, after the café closed, sugar shakers were weighted to assess the remaining sugar. For weighing, a standard kitchen scale measuring to an accuracy of 1 g was used. To determine sugar intake per hot beverage (in g), the amount of used sugar was divided by the number of hot beverages registered as sold in the cashier system. Hot beverages included the café’s typical selection of drinks such as various coffee products (i.e., coffee, café crème, cappuccino, latte macchiato, espresso, milk coffee) and hot chocolate, chai latte, and tea.

#### Customer questionnaire

Customers used a 7-point Likert scale to evaluate the intervention on four different dimensions (*effective, helpful, annoying, adequate*), following Kroese et al. [12] and assessed the strength of perceived influence on personal sweetening behavior, i.e., perceived lack of agency (1-'not at all' to 7-'strongly'). In addition, demographics, i.e., age, gender, self-reported height and weight as well as professional status, were also assessed.

### Analytical procedure

To compare default and nudging condition a t-test for independent samples was conducted. The effect size was calculated following Rasch, Friese, Hofmann, and Naumann [13]. All analyses were conducted with IBM SPSS Statistics (version 25).

## Results

### Sugar use

Of the 17,233 hot beverages and 12,589 g of sugar that were used in total, 8533 hot beverages and 6887 g of sugar were used in the default condition and 8700 hot beverages and 5702 g of sugar in the nudge condition.

**Fig. 2:**
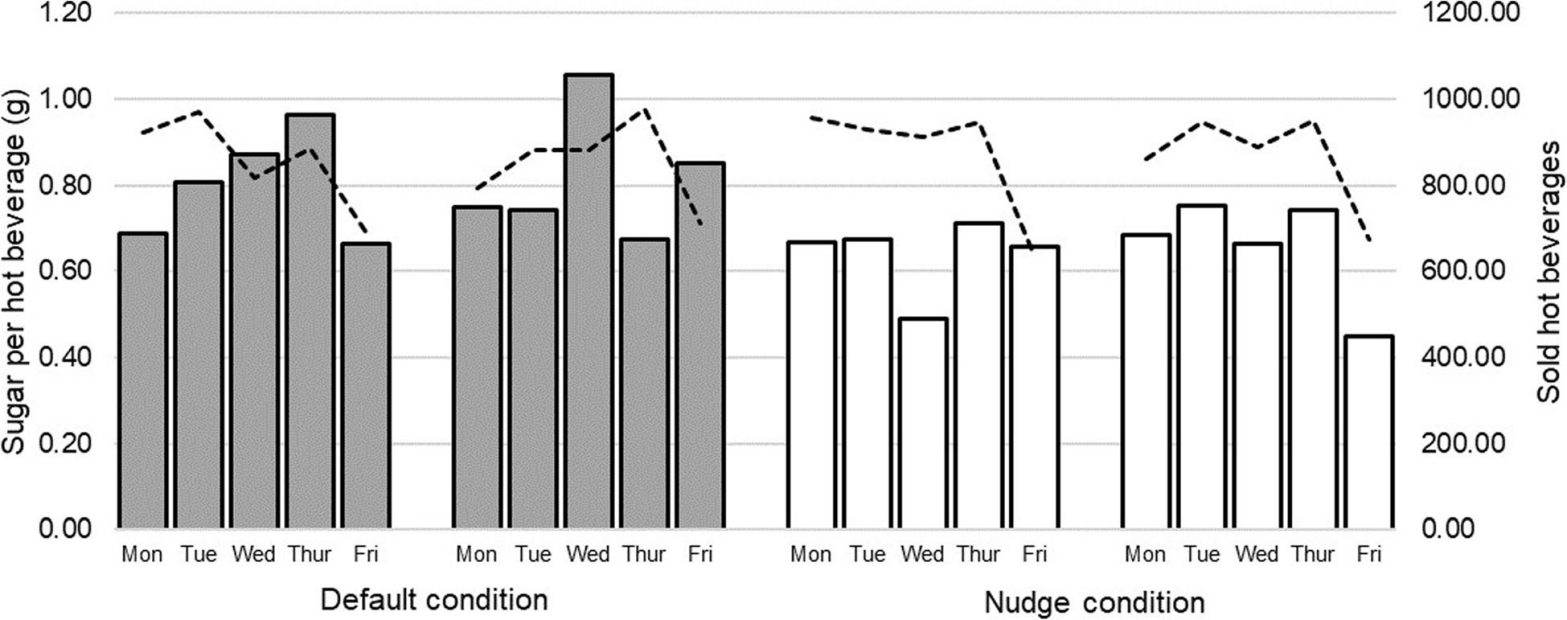
Sugar use per hot beverage during the default and nudge conditions (bars) and number of sold beverages (lines)

An average of 0.81 g (*SD* = 0.13) of sugar was used per hot beverage in the default condition, ranging between 0.66 g and 1.06 g per day. This dropped to an average of 0.65 g (*SD* = 0.10) per hot beverage in the nudge condition, ranging from 0.45 g to 0.75 g per day. Comparing the use of sugar per hot beverage between conditions (see Fig. 2) resulted in a significant difference of 0.16 (*t*(14)  = 3.02; 95% *CI* = 0.05–0.27; *p* = 0.007), which indicates a strong effect of *d* = 1.35. This means that during the nudge condition, sugar use was reduced by 19.75% as compared to the default condition.

### Acceptance of the intervention

A total of 59 customers (62% female; 78% students) with a mean age of *M* = 26 years (*SD* = 7.3) and an average BMI of *M* = 22.4 kg/m^2^ (*SD* = 3.6) evaluated the intervention at the end of the study. They regarded the intervention as a helpful (*M* = 5.56, *SD* = 0.99), effective (*M* = 5.39, *SD* = 1.27), and adequate (*M* = 5.29, *SD* = 1.22) strategy for reducing sugar consumption. Furthermore, both annoyance with the intervention (*M* = 2.83, *SD* = 1.90) and perceived lack of agency were rated as low (*M* = 2.36, *SD* = 1.99).

## Discussion

The present findings, that were derived from a real-world experiment, show that making minor changes to daily food environments can lead to positive effects on sugar intake in habitual behaviors. Modifying the functional design of sugar shakers resulted in a reduction of the amount of added sugar of 19.75% compared to the default condition, which strengthens the results on table salt use [7–10, 15] and adds to the evidential basis that reducing dispensed quantities can sustainably change individual behaviors in public places.

Data shows that the reduction in sugar use during the nudge condition was considerably stable across the assessment days (see Fig. 2). Three possible mechanisms might have contributed to this reduction. Firstly, smaller amounts per pour might allow for a more precise regulation of the amount of sugar being added, which could in turn have helped customers to better self-monitor their consumption behavior. Secondly, as customers had to use the modified sugar shaker more often to get the same amount of sugar as in the default condition, pouring might have induced a greater attention and awareness of the amount of sugar they were adding. The influence of cognitive processes such as attention and awareness on food intake has been demonstrated in a review across 24 studies [16]. Thirdly, as adding sugar to every drink is highly habitual, it might be that people pour once or twice out of routine, regardless of the amount that is distributed per pour. In line with this notion, Greenfield et al. [7, 8] concluded that consumers used the same manual actions with different salt pots. However, Farleigh et al. [9] compared the salt shakers with three different hole sizes (large, medium, small) and found widely different shaking times suggesting that consumers were more aware of the amount added and/or were more deliberately monitoring their pouring behavior. Interestingly, similar as in the present study, smaller hole sizes reduced the amount of added salt consistently across the 10 intervention days compared to the other conditions despite longer shaking times. Thus, consumers did adapt their behavior but not to the degree that it returned to the baseline level of use. Also the lack of learning effects, i.e. indicated by an increase of added sugar over time in the nudging condition, suggests that many consumers added sugar before they tasted the beverage. Adding sugar before tasting implies a considerable influence of habit on sugar use rather than adding to individual preference (cf. also Farleigh et al., [9]). Specifically take-away settings, as in the present study, might facilitate habitual behavior (such as adding salt or sugar before tasting) due to time constrains. In addition, to gain more certainty about the effect it would be important that future studies replicate findings in other cafés and expand the intervention to additional settings where sugar shakers are available for individual sweetening of hot beverages such as sit-down cafés and larger cafeterias.

In line with previous research that focused on the food environment (for similar results see Kroese et al. [12]; Van Gestel et al. [17]), the present intervention was evaluated positively, and acceptance rates were high. In contrast, people are generally more resistant towards directive and restrictive strategies such as taxation and prohibitions [14, 18]. Besides positive evaluations, recent research and policy proposals emphasize the potential of changing the food environment to improve eating behavior [19, 20], highlighting the fact that individual control over actions and decisions is overestimated while the influence of the food environment is underestimated [20, 21]. The present results support the notion that public interventions targeting the environment by functionally modifying everyday objects appear highly promising.

Even though the results of the present study showed a reduction of 19.75% per beverage, reducing sugar intake in hot beverages is only one component towards the goal of reducing sugar overconsumption. In addition, as we focused on hot beverages and not the entire diet it remains unclear if customers (over-)compensated their lower sugar use in subsequent intakes. Therefore, to gain a comprehensive picture of the overall effect the entire diet needs to be taken into account. Furthermore, to reduce sugar overconsumption effectively, the proposed intervention strategy must also be complemented by other approaches that tackle different areas of daily sugar consumption [20, 22]. Nevertheless, a gradual reduction of added sugar contains the potential to reduce overweight, adiposity, and type-2-diabetes prevalence [23], and a general implementation of the present results in public life can act as a first step in support of this endeavor.

## Conclusion

The present study provides evidence for a simple, cost-effective, and widely applicable intervention strategy in public places, highlighting that even minor changes in food environments can lead to positive effects. An implementation of the present results in public life can therefore be a first step towards conquering the problem of sugar overconsumption.

## Data Availability

The datasets analyzed during the current study are available from the corresponding author on reasonable request.
